# Modelling the Northward Expansion of *Culicoides sonorensis* (Diptera: Ceratopogonidae) under Future Climate Scenarios

**DOI:** 10.1371/journal.pone.0130294

**Published:** 2015-08-24

**Authors:** Anna Zuliani, Alessandro Massolo, Timothy Lysyk, Gregory Johnson, Shawn Marshall, Kathryn Berger, Susan Catherine Cork

**Affiliations:** 1 Department of Ecosystem and Public Health, Faculty of Veterinary Medicine, University of Calgary, Calgary, Alberta, Canada; 2 O'Brien Institute for Public Health, Cumming School of Medicine, University of Calgary, Calgary, Alberta, Canada; 3 Agriculture and Agri-Food Canada, Lethbridge Research Centre, Lethbridge, Alberta, Canada; 4 Department of Animal & Range Sciences, Montana State University, Bozeman, Montana, United States of America; 5 Department of Geography, University of Calgary, Calgary, Alberta, Canada; The Pirbright Institute, UNITED KINGDOM

## Abstract

Climate change is affecting the distribution of pathogens and their arthropod vectors worldwide, particularly at northern latitudes. The distribution of *Culicoides sonorensis* (Diptera: Ceratopogonidae) plays a key role in affecting the emergence and spread of significant vector borne diseases such as Bluetongue (BT) and Epizootic Hemorrhagic Disease (EHD) at the border between USA and Canada. We used 50 presence points for *C*. *sonorensis* collected in Montana (USA) and south-central Alberta (Canada) between 2002 and 2012, together with monthly climatic and environmental predictors to develop a series of alternative maximum entropy distribution models. The best distribution model under current climatic conditions was selected through the Akaike Information Criterion, and included four predictors: Vapour Pressure Deficit of July, standard deviation of Elevation, Land Cover and mean Precipitation of May. This model was then projected into three climate change scenarios adopted by the IPCC in its 5^th^ assessment report and defined as Representative Concentration Pathways (RCP) 2.6, 4.5 and 8.5. Climate change data for each predictor and each RCP were calculated for two time points pooling decadal data around each one of them: 2030 (2021–2040) and 2050 (2041–2060). Our projections showed that the areas predicted to be at moderate-high probability of *C*. *sonorensis* occurrence would increase from the baseline scenario to 2030 and from 2030 to 2050 for each RCP. The projection also indicated that the current northern limit of *C*. *sonorensis* distribution is expected to move northwards to above 53°N. This may indicate an increased risk of Culicoides-borne diseases occurrence over the next decades, particularly at the USA-Canada border, as a result of changes which favor *C*. *sonorensis* presence when associated to other factors (i.e. host and pathogen factors). Recent observations of EHD outbreaks in northern Montana and southern Alberta supported our projections and considerations. The results of this study can inform the development of cost effective surveillance programs, targeting areas within the predicted limits of *C*. *sonorensis* geographical occurrence under current and future climatic conditions.

## Introduction

The geographic ranges of arthropod vectors and the diseases they transmit, are determined by host and virus availability under suitable climatic and environmental conditions [1]. Climate change is expected to have an impact on the distribution of many arthropod vectors with greater responses likely at higher latitudes [2, 3].

Vector distribution modeling has been used to assess the risk of emerging and re-emerging vector-borne disease incursions and for planning disease prevention, surveillance and vector control measures (e.g. [4]).

Amongst the wide range of arthropod disease vectors, *Culicoides* biting midges are believed to be particularly responsive to global climate change [5, 6]. *Culicoides* species have the potential to transmit several pathogenic viruses to wild and domestic ungulates [7]. Two of the most important pathogens of ungulates in North America are the Orbiviruses that cause Bluetongue Disease (BT) and Epizootic Hemorrhagic Disease (EHD). These diseases can cause high morbidity and mortality resulting in significant economic loss for the livestock industry as well as impacts on the wildlife recreation and hunting sector due to their dramatic impact on wild populations. Bluetongue and EHD are transmitted by *Culicoides sonorensis (Diptera*: *Ceratopogonidae)* (Wirth and Jones) in the western United States, where both diseases are endemic [8]. Orbiviruses are currently not endemic in Canada, despite sporadic virus incursions of BTV and EHDV [9] in the Okanagan Valley of British Columbia and EHD outbreaks in Alberta in 1962 and in 2013 [10, 11]. In 2013 both viruses were isolated in the two westernmost provinces of Canada (EHDV in Alberta and BTV in British Columbia) raising the concern that the risk of orbivirus transmission might increase in the region [11–14]. As a result of these concerns there is a growing need to enhance vector surveillance programs in Canada. Modeling the current and future distribution of *C*. *sonorensis* will help to develop targeted vector surveillance programs, thus contributing to EHD and BT control in this region.

Previous work to model *Culicoides* spp. distribution under current and future climate conditions has been done in Europe as a result of the emergence of BTV into that continent [15–17]. In North America, the factors influencing the distribution of *C*. *sonorensis* have not yet been fully evaluated, but a few studies have made an attempt to link climate and environmental factors to vector or disease occurrence [18–20]. The latter studies, together with the work done specifically on *C*. *sonorensis* biology [18, 21–25], have helped our understanding of the ecology of *Culicoides sonorensis* in North America.

In this study we integrated field information on *C*. *sonorensis* presence with climate and environmental data and used a machine learning approach to model the complex relationship between vector presence and large scale proxies. Among all the available algorithms, the maximum entropy method [26] was selected. This method provides a powerful presence-only approach able to construct a reliable model of the potential distribution of a species using the relationship between species presence records and the climate and environmental characteristics at presence sites (without assuming species absence in unsampled locations) [27].

The objectives of this study were then (a) to identify environmental proxies to predict *C*. *sonorensis* presence at large scale; (b) to model current *C*. *sonorensis* distribution at the edge of its distribution in south-central Alberta (Canada) and Montana (USA); and (c) to project the northern limit of the distribution of *C*. *sonorensis* in short-term (2030, pooling 2021–2040 data) and mid-term (2050, pooling 2041–2060 data) climate change scenarios. The implications and applications of such projections for *Culicoides*-borne disease incursions in the north-west of Canada are discussed.

## Materials and Methods

No animal care was required as the subjects were invertebrates. No permits were required as field work was conducted on private land with the permission of the owners.

### Study area

**Fig 1:**
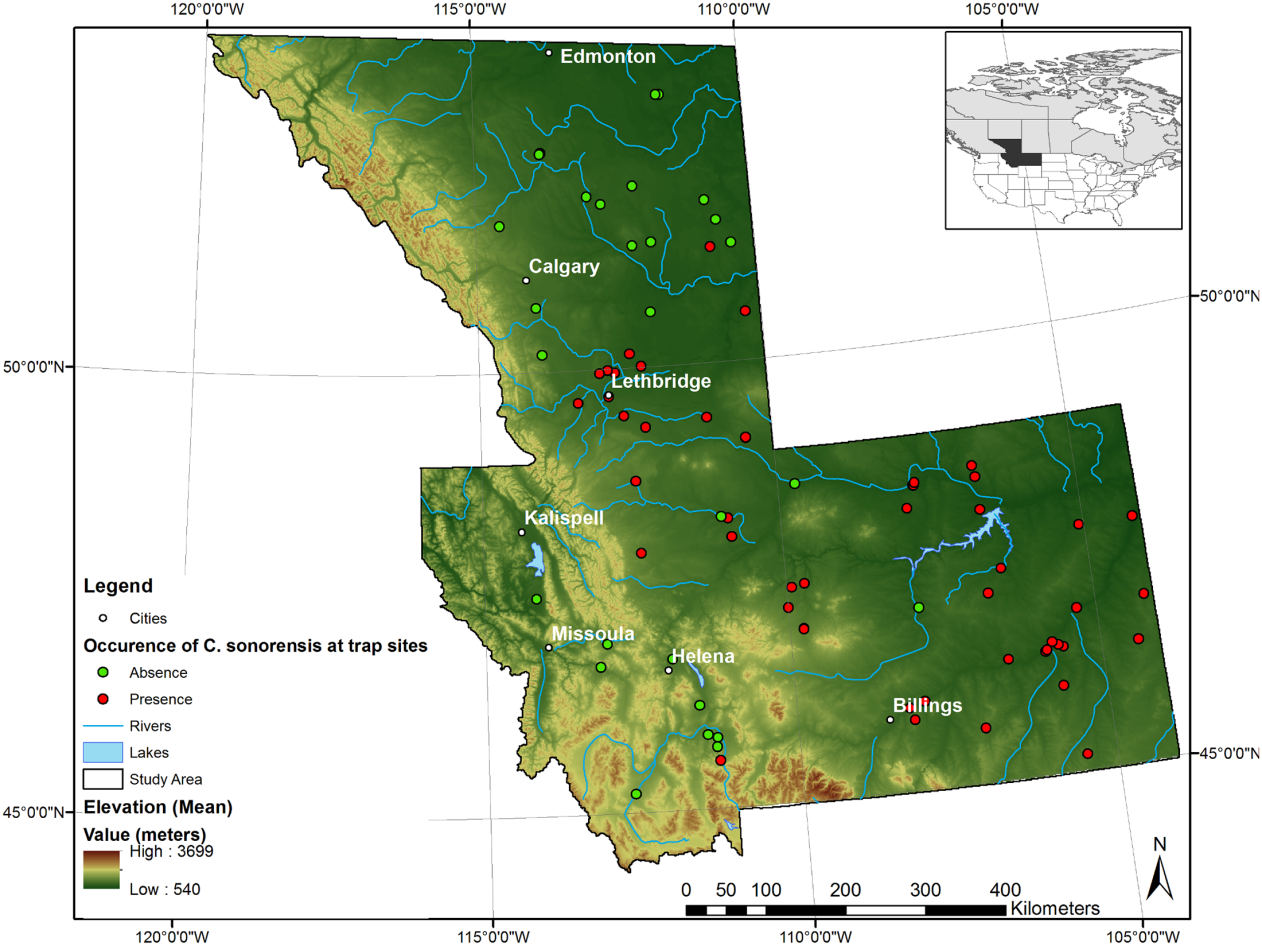
Topographic map of the study area southern Alberta (CAN) and Montana (USA) where *Culicoides sonorensis* presence was investigated from 2002 to 2011.

The study was carried out using entomological data from south-central Alberta, Canada and Montana, USA (Fig 1), an area spanning from the 44^th^ to the 54^th^ parallel North and from the 104^th^ to 120^th^ meridian West, measuring 618,337 km^2^.

Three ecological regions, the Northern Forests, North Western Forested Mountains and Great Plains (Northwestern Great Plains and Northwestern Glaciated Plains [28]), characterize the environment type of the study area. The Northern Forests ecoregion consists of hilly terrain and has long, cold winters and short summers. Mean summer temperatures range between 11°C and 18°C. In the western part of the ecoregion (the easternmost in the study area defined above) mean winter temperature is -25.5°C and mean annual precipitation is 400 mm. Northwestern Forested Mountains are associated with extensive mountains separated by wide valleys and lowlands. The climate is sub-arid to arid and mild in southern valleys and humid and cold at higher elevations. Mean summer temperatures range between 10°C and 21°C. Winter temperatures range between -23°C and 0°C. Annual precipitation varies with the elevation and ranges between 250 mm to 500 mm. Great Plains were once covered with natural grassland and are now partially replaced by rangeland and croplands. The climate is semi-arid and continental, characterized in the north by short, hot summers with mean temperatures of about 15°C and long, cold winters (mean -12.5°C) with periodic droughts and frosts [29].

The elevation (mean value per Km^2^) ranges from 545 m to 3,699 m (DEM, DMTIspatial via University of Calgary). The main rivers in the region are the South Saskatchewan River in southern Alberta and the Missouri and Yellowstone rivers in Montana.

### Entomological data

*Culicoides* trapping was conducted using miniature downdraft blacklight traps (J.W. Hock Inc., Gainesville, FL, US). Data on the presence of *C*. *sonorensis* midges were collected in south-central Alberta (TL) and Montana (GJ) over five years (2002, 2003, 2009, 2010, 2011) and four years (2002, 2003, 2008, 2011), respectively (S1 Table).

The purpose of the sampling was different for Alberta and Montana. Collections in south-central Alberta were designed to study abundance patterns and species composition at cattle feedlot locations and resulted in a comprehensive entomological survey where traps were monitored throughout the vector season [19, 30, 31]. In Montana, traps were set during vector seasonal peaks as part of a BTV surveillance pilot project that aimed to confirm the presence of *C*. *sonorensis* in the state, with a special focus on high risk areas for BTV transmission (GJ, personal communication).

Seventy-eight trap sites, 30 in south-central Alberta and 48 in Montana were identified during the study period 2002–2011, ranging in latitude from 53°N in Alberta to 45°N in Montana, and in longitude from 111°W to 105°W (Fig 1). Certain sites were resampled between 2002 and 2011 (Table 1). For modelling vector presence at the edge of its range, a site was considered a presence point for *C*. *sonorensis* if a female specimen was captured at least once at that specific location throughout the study period. Fifty trap-sites, 14 in south-central Alberta and 36 in Montana were considered presence sites for *C*. *sonorensis* and were used to build the distribution model.

**Table 1:** Entomological sampling effort for the detection of *Culicoides sonorensis* from 2002 to 2011 in Alberta (AB, Canada) and Montana (MT, US). In table are reported the name of the sampled area (Location), the source of information (data from T. Lysyk or G.D. Johnson), the year of collection (Year), the number of sites in each area (Nr. of sites), duration (weekly, sampling for a week; nightly, sampling for one night only), trapping effort (number of trap nights: number of traps * number of nights), and the number of sites in which *C*. *sonorensis* was recorded (positive).

Province/State	Site	Source	Year	Nr. of sites	Duration	Samples	Trap Night	Positive
Alberta/Canada	AB– 1	Lysyk 2006 [30]	2002	8	Weekly	18–22	126–154	6
			2003	8	Weekly	23–25	161–175	8
Alberta/Canada	AB– 2	Lysyk and Dergousoff [19]	2009	7	Weekly	20–21	140–147	3
Alberta/Canada	AB– 3	Lysyk and Dergousoff [19]	2010	10	Weekly	19–20	133–140	7
			2011	10	weekly	17–19	119–133	6
Alberta/Canada	AB– 4	Lysyk and Dergousoff [19]	2011	9	Weekly	2	14	0
Montana/US	MT– 1	Johnson (unpubl.)	2002	31	Nightly	1–9	3–27	26
Montana/US	MT– 2	Johnson (unpubl.)	2003	17	Nightly	5–10	10–20	15
Montana/US	MT– 3	Johnson (unpubl.)	2008	8	Nightly	4–8	4–8	7
Montana/US	MT– 4	Johnson (unpubl.)	2011	12	Nightly	6 *	6*	5

*One site (positive) was sampled once.

### Environmental data

We selected environmental variables (Table 2) as proxies of *C*. *sonorensis* presence on the basis of previous studies on *Culicoides* species distribution modelling [32–35] and *Culicoides* spp. ecology [23, 36, 37]. Each proxy was calculated at 1 x 1 km resolution, which was the finest achievable resolution considering the available data and the large extent of the study area.

**Table 2:** Variables used to characterize habitat for *Culicoides sonorensis* presence in Alberta (Canada) and Montana (US).

Variable	Description	Source
**Elevation (E)**	Mean, Minimum, Maximum, Standard deviation (m)	DEM, University of Calgary
**Aspect (A)**	Categorical	DEM, University of Calgary
**Land cover (LC)**	Reclassified in 7 Classes: Water Bodies, Evergreen Needle Leaf Forests, Open Shrubland, Grassland, Cropland, Barren or Sparsely Vegetated and Others	MODIS (2004)
**Temperature (*T*)**	Monthly means for May–October (K)	NARR* (1991–2010)
**Relative humidity (*RH*)**	Monthly means for May–October (%)	NARR* (1991–2010)
**Precipitation (*P*)**	Monthly means for May–October (mm)	NARR* (1991–2010)
**Vapour pressure deficit (*VPD*)**	Monthly means for May–October (KPa)	Computed using July *T* and *RH* from NARR* (1991–2010)
**Normalized Difference Vegetation Index (NDVI)**	Mean and standard deviation for May-October	MODIS (2002–2011)
**Middle infrared radiation (MIR)**	Mean and standard deviation for May-October	MODIS (2002–2011)

* = North American Regional Reanalysis project (NARR).

Primary terrain attributes (i.e., elevation and aspect) were obtained from a 30 m resolution Digital Elevation Model (DEM, DMTIspatial via University of Calgary). Elevation (mean, minimum, maximum and standard deviation) and aspect (4 classes: North-East, South-East, South-West and North-West) were calculated directly from digital elevation data, and used to provide information on the geomorphology of the landscape.

Land Cover, Normalized Difference Vegetation Index (NDVI) and Middle Infrared Radiation (MIR) values at 1 km² resolution were derived from the MODerate Resolution Imaging Spectroradiometer (MODIS-Terra, Vegetation Indices Monthly L3 Global 1km, product ID MOD13A3; https://lpdaac.usgs.gov/products/modis_products_table/mod13a3) instrument operating on Terra spacecraft and distributed by the Land Processes Distributed Active Archive Center [38]. NDVI was used as a proxy of plant growth, vegetation cover and biomass production and is often correlated with soil moisture and rainfall [39]. MIR is a subregion of the infrared (IR) wave length and was used as a proxy of water content, surface temperature, and tree canopy density and structure [39]. Cumulative NDVI and MIR monthly means and standard deviations for each month of our sampling season (May-October) were calculated over a 10 year period (2002–2011), which was the longest available using MODIS data (product ID MOD13A3).

The Land Cover Type 1 dataset is a yearly product (NASA Land Processes Distributed Active Archive Center (LP DAAC); MODIS-Terra, Land Cover Type Yearly L3 Global 500m/1Km—product ID MOD12Q1, 2004; https://lpdaac.usgs.gov/data_access) with 17 land cover classes identified by the International Geosphere Biosphere Programme (IGBP).We grouped the 17 classes into 7 land cover classes considering the role that they play in *Culicoides* spp. habitat suitability (S2 Table). The new pooled classes included water bodies (class 1), evergreen needle leaf forests (class 2), open shrubland (class 3), grassland (class 4), cropland (class 5), barren or sparsely vegetated (class 6) and others (class 7).

### Climate data

Monthly mean temperature (*T* in °C), relative humidity (*RH* in %) and precipitation (*P* in kg/m^2^/s and then converted in mm/month) from May to October were obtained at approximately 0.3 degrees resolution from the U.S. National Oceanic and Atmospheric Administration (NOAA) National Centers for Environmental Prediction (NCEP) within the NARR (North America Regional Reanalysis) project (http://www.esrl.noaa.gov/psd/data/gridded/data.narr.html). Vapour pressure deficit (*VPD*) is a measure of the drying power of the air and it is an important proxy for insect survival [40]. *VPD* is calculated as the difference between the saturation vapour pressure, *e*_*s*_, calculated from the temperature, and the actual vapour pressure, e_v_, calculated from *e*_*s*_ and *RH* [41]. Monthly means for *T*, *RH*, *VPD* and *P* were calculated by pooling monthly data for each variable for a twenty year period (1991–2010) to describe the climate of the study area. Layers for each variable were computed at 1 km^2^ resolution using an ordinary kriging interpolation technique, as implemented in ArcMap 10.1.

For the climate change scenarios, we considered three Representative Concentration Pathways (RCP) which describe future greenhouse gas emissions and associated radiative forcing on the basis of different trends in climate change drivers such as global population, economic development, energy systems, and land-use change [42]. We examine scenarios for RCP 2.6, RCP 4.5 and RCP 8.5, which correspond to maximum 21^st^ century atmospheric radiative forcings of 2.6, 4.5 and 8.5 W/m^2^ [43]. These represent low, moderate, and high future warming scenarios and correspond to aggressive, moderate, and minimal implementation of climate policy to reduce greenhouse gas emissions, as designed for the IPCC fifth assessment report on future climate change [44]. These scenarios bracket the range of anthropogenic climate forcing that is expected this century.

Projected future values for climate variables were obtained from the Fourth Generation Canadian Coupled General Circulation Model (CanESM2) [44]. CanESM2 has been developed by the Canadian Centre for Climate Modeling and Analysis (CCCma) following the reliability criteria defined by the IPCC [44–46]. Climate change outputs for each RCP scenario were extracted for two time periods: 2030, 2021–2040, and 2050, 2041–2060.

Since CanESM2 outputs, like all General Circulation Models (GCM), are at a coarse resolution (i.e., grid spacing approximately 2.81 degrees in longitude and 2.79 degrees in latitude), historical data (1991–2010) were obtained from the same GCM and for the same predictors to build climate layers as ‘perturbations’ to observed (NARR) climatology [38], following the IPCC guidelines [44]. Thus, more reliable layers were built by computing the difference (or the ratio in the case of July *VPD*) between the CanESM2 projected variables and their CanESM2 baseline values. This difference/ratio was added as a perturbation to the NARR reference climatology for the future scenarios.

### Statistical Modeling

#### Model formulation

We used the maximum entropy algorithm for presence-only data, implemented through MaxEnt software version 3.3.3k [47], to model species distribution. It was selected because of the lack in homogeneity in temporal and spatial sampling effort between data from Alberta and Montana. This lack in data homogeneity made inappropriate any attempt to look at the two databases in terms of species absence records and seasonal patterns.

MaxEnt software produces a model of the species probability distribution with maximum entropy subject to environmental constrains, called features, which ensure a solution as close as possible to reality. The study area is the space where the MaxEnt probability distributions are defined. The main focus of MaxEnt is to maximize the entropy of the distribution, minimizing the relative entropy or information gain, a measure of goodness of fit which is obtained for each iteration of the model. The maximum entropy distribution is defined as the ratio between the probability density distribution of environmental variables where the species is present *f*_*1*_ *(x)* over the probability density distribution of environmental variables across the study area *f(x)* [47]. A regularization parameter lambda (*λ*) has been introduced to prevent MaxEnt of matching the empirical feature means too closely to the real feature means from the study area providing complex models not appropriate for generalization. Default *λ* parameters in MaxEnt were tuned over presence points of an international dataset covering six geographic regions [27, 48]. The best model would be the one that maximizes the difference between the log likelihood and the regularization, or in other words, the one that minimizes the relative entropy subject to the error bound constraints, balancing model fit and complexity [27].

The algorithm was run for the variables at current climatic conditions with five replicates, 500 iterations and 10,000 random background points. MaxEnt does not assume these to be absence points, but as a range of environmental conditions where the vector could potentially be present. The choice of the number of replicates was higher than the default settings (i.e. 1) and was balanced between the search for stable outputs and the need to run the models within reasonable timeframes. Iterations and background points were set as default. All the climatic and environmental variables were transformed into hinge feature that combines linear and step functions and this improves model performance when there are at least 15 presence points [48].

The contribution of different predictors was estimated using a measure of their contribution to the model, and their permutation importance. The contribution of each variable to the model is calculated as the increasing training gain due to the variable in each iteration of the model.

Variables that had less than 1% contribution to the increasing training gain or less than 1% permutation importance [49] were considered unimportant and excluded from the subsequent analyses. Permutation importance is measured as the difference in accuracy when the final model is compared to one in which values of the considered variable at presence locations and background points are permutated [50]. The variables were tested for cross-correlation by calculating the Pearson correlation coefficient (*r*) using the raster statistic calculator implemented in ENMtools [51]. Competing models were built using all the proxies and then reducing model complexity by alternatively removing highly correlated variables (*r* > 0.8). Because of the high correlation values among *VPD*, *RH* and *P* (*VPD* is actually calculated from *RH*, *T* and *P*) and because of their high ranking in the contribution and permutation values three additional MaxEnt models were built using respectively: (a) all variables excluding *RH* and *T* since they were used to calculate *VPD*; (b) all variables except *VPD* and *RH* to evaluate the strength of *P*; and (c) all variables except *VPD* to evaluate the strength of *RH*. We formulated the final models only with those predictors that were not strongly correlated (*r* < 0.5). For each alternative model, 80% of the occurrence points were used for training the model and 20% were set apart to test its accuracy.

The spatial distribution of the model outcome was obtained by reclassifying and mapping the probability levels into five classes of probability of occurrence: class 1 (0–0.2, very low probability), class 2 (0.21–0.4, low probability), class 3 (0.41–0.6, moderate probability), class 4 (0.61–0.8, moderate-high probability) and class 5 (0.81–1, high probability).

#### Model selection

We compared the accuracy of competing models by Receiver Operating Characteristics (ROC) curves, a threshold-independent method that combines sensitivity and specificity [52]. We also compared different models using the Akaike Information Criterion (AIC; [53]) and selected the "best performing" model using a modified AIC with a correction term for sample size, AIC_C_ using ENMTool [51]. For the best model, response curves describing the relationships between the variation in individual proxies and the probability of *C*. *sonorensis* occurrence were examined to study the effect of each predictor on the presence of the species.

#### Model application under climate change scenarios

The climate variables of the best model developed for the current conditions were then projected onto the future climatic conditions. Ten replicates and 500 iterations of the MaxEnt algorithm were run for each pathway (i.e. RCP 2.6, RCP 4.5 and RCP 8.5) and time point (short-term and mid-term scenarios) with the same model settings. We decided to run more replicates with climate change scenarios compared to the model under current climatic condition to get predictions as stable as possible, especially considering the uncertainties of climate change scenarios.

The RCP climate scenarios include land-cover change scenarios, with respect to e.g. shifts in cropland, forest harvest, vegetation cover, and urban development [42, 43, 54]. A particular climate model may include additional land-use/land-cover feedbacks, depending on the interactive land surface model in the future climate change scenario. We limit our study to these internal land-use change scenarios and feedbacks within the climate projection, and do not make additional assumptions about land cover change in our study area.

Moreover, we cannot easily remap the RCP and CanESM2 land cover treatment into the historical land cover classifications that we use. We therefore assume no change in land cover for the 2021–2040 and 2041–2060 projections, other than those implicit in the climate projections. Regional climate change impacts are likely to far exceed those associated with land-cover change over this time scale, although land cover changes can be important locally. Our results for 2021–2040 and 2041–2060 are limited to the effects of shifts in climate.

## Results

### Model selection

In the first MaxEnt run, 11 environmental variables achieved more than 1% in both contribution and permutation importance. Cross-correlation was investigated for 10 variables; Land Cover was not included because it is a nominal predictor. Correlation values (*r*) for the selected variables are shown in Table 3. Highly correlated variables (*r*> 0.8) included *VPD* of July, *RH* of July and August and *P* of August. Other correlated variables were *T* of October and *VPD* of July (*r = 0*.*78*) and *P* of May and *NDVI* of October (*r = 0*.*56*). Because of the consistently high contribution and permutation importance of *VPD* of July (43.9% and 32.1% respectively) and *RH* of July (46.9% and 23.9% respectively), these variables were considered key predictors for *C*. *sonorensis* presence and used to build the final models (Table 4). On the other hand, because of the high correlation of *P* and *RH* of August with *VPD* and *RH* of July, and their overall lower performance, these variables were excluded from subsequent analyses. Nine of the 11 variables that scored above 1% on both contribution and permutation importance in the first MaxEnt run were considered to build the final models. Five final models were built using sub-groups of the nine selected variables on the basis of their biological importance and their cross-correlation values (Table 4).

**Table 3:** Correlation matrix showing Pearson correlation coefficients (*r*) for the 10 predictors that obtained more than 1% contribution and permutation importance in the first preliminary run of MaxEnt. Asterisks indicate highly correlated variables (r > 0.5).

Variables	NDVI Aug sd	NDVI Oct mean	NDVI Oct sd	*P* Aug	*P* May	*RH* Aug	*RH* Jul	*T* Oct	*VPD* Jul
E sd	-0.373	0.326	0.351	0.065	0.444	0.282	0.261	0.059	-0.232
NDVI Aug sd		-0.377	0.068	-0.045	-0.324	-0.166	-0.140	0.138	0.103
NDVI Oct mean			0.169	0.366	**0.560**	0.494	0.490	0.195	-0.471
NDVI Oct sd				0.235	0.279	0.258	0.245	0.119	-0.243
*P* Aug					0.369	**0.789**	**0.764**	0.167	**0.836**
*P* May						0.419	0.421	0.452	-0.315
*RH* Aug							**0.984**	**0.089**	**-0.953**
*RH* Jul								0.108	**-0.955**
*T* Oct									**0.782**

In **bold** are reported highly correlated variables (r > 0.5).

**Table 4:** Best models developed with MaxEnt predicting *Culicoides sonorensis* presence in Alberta (Canada) and Montana (US). Table includes variables included into each best mode (A–E). Variables, percent contribution, permutation importance, corrected AIC, delta (Δ) and Akaike weights (ω) are provided for each best fit model.

Model	Training data AUC	Testing data AUC	Variables	% Contribution	Permutation importance	AICc	Δ	ω
**A**	0.84	0.76	*RH* Jul	59.7	70.2	1319.99	13.610	0.001
			E sd	22.1	12.9			
			LC	12.3	3.5			
			*P* May	3.6	8			
			*T* Oct	2.3	5.4			
**B**	0.83	0.74	*VPD* Jul	54.5	72	1306.38	0	0.994
			E sd	27.5	17.3			
			LC	14.9	2.7			
			*P* May	3	8			
**C**	0.85	0.79	*RH* Jul	55.1	62.6	1316.93	10.550	0.005
			E sd	20.9	11			
			LC	12.9	6.5			
			NDVI Oct m	9.1	17.1			
			*T* Oct	2	2.7			
**D**	0.85	0.76	*VPD* Jul	47.4	44.1	1330.37	23.989	0.000
			E sd	25.4	28.9			
			LC	13.5	5.8			
			NDVI Oct sd	5.6	7.5			
			NDVI Aug sd	4.4	8.1			
			NDVI Oct m	3.7	5.6			
**E**	0.82	0.76	*RH* Jul	62	66.3	1363.41	57.027	0.000
			E sd	23.1	19.6			
			LC	12.7	4.7			
			*T* Oct	2.2	9.4			

All of the models had high accuracy (AUC > 0.70) and high overall performance (% of correctly classified presence cases), to select the best model among the final five, we used the sample size corrected Information Criterion (AICc). Model B had the lowest AICc (Table 4) and was therefore selected as the best model for describing *C*. *sonorensis* current distribution and to be projected under future climate scenarios.

**Fig 2:**
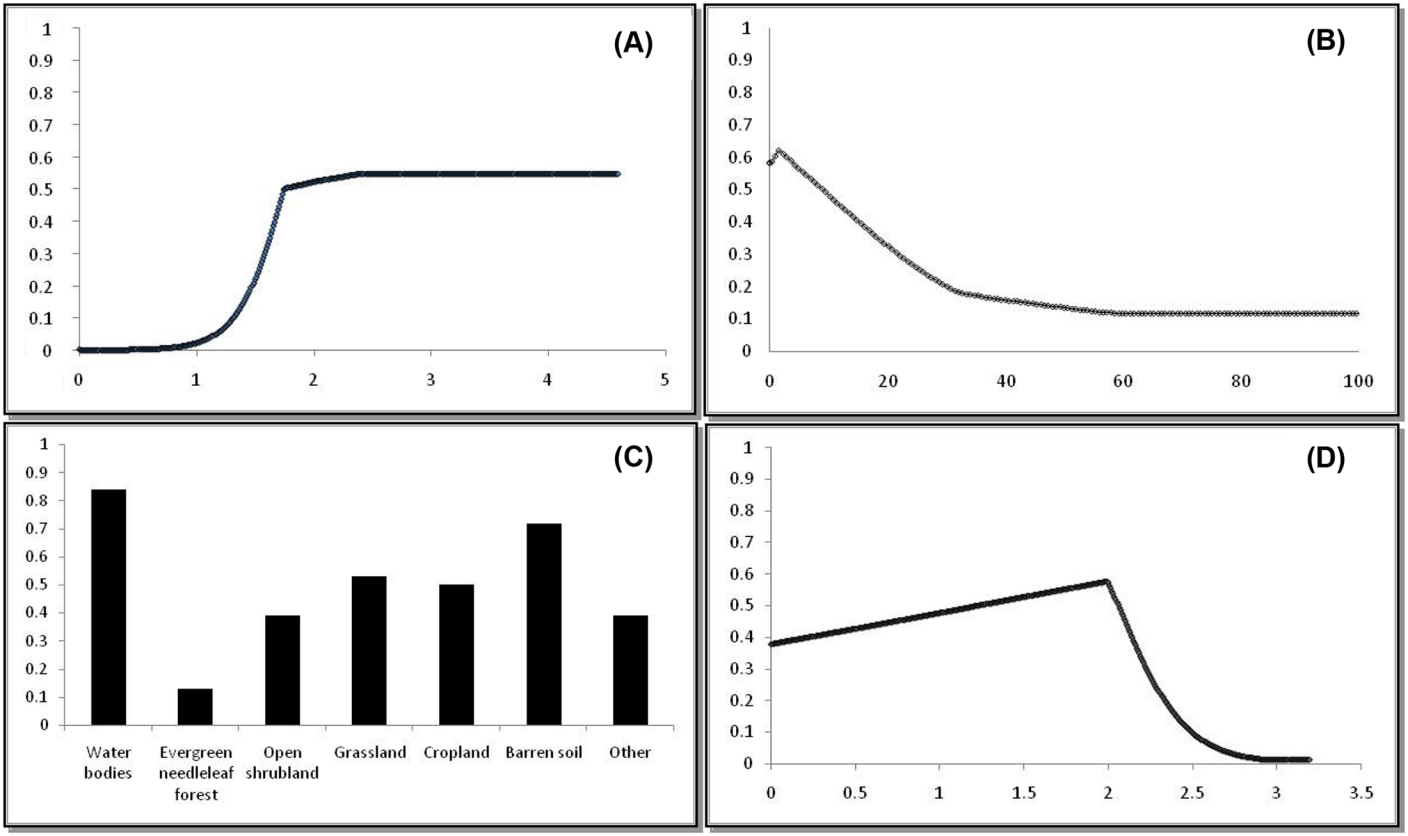
Response curves plotting the probability of *Culicoides sonorensis* against the values of top ecological predictors. (A) vapour pressure deficit of July (; (B) standard deviation of elevation; (C) Land Cover; (D) precipitation of May. The x-axis represents the variable value and the y-axis represents the probability of *Culicoides sonorensis* presence as estimated by best MaxEnt model.

The relative contribution of the explanatory variables to model B (S1–S4 Figs) showed that *VPD* of July contributed the most (up to 54.5%) followed by E SD, LC and *P* of May (respectively 27.5%, 14.9% and 3%). *VPD* of July showed an increased probability of *C*. *sonorensis* occurrence with increasing values of *VPD* reaching a plateau above 1.7 kPa (Fig 2).

The highest probability of *C*. *sonorensis* presence (*P* > 0.6) was observed with low elevation heterogeneity. When the variation of elevation (Esd) exceeded 60m within the same pixel there was a minimal predicted probability of *C*. *sonorensis* occurrence. Water bodies (class 1) and barren terrains (class 6) were positively associated with *C*. *sonorensis* occurrence. Open shrublands (class 3) were associated with a 0.38 probability of *C*. *sonorensis* occurrence, whereas grasslands and croplands (class 4 and 5) predicted *C*. *sonorensis* presence no better than random. A strong negative correlation was detected between needle-leaf forests (class 2) and *C*. *sonorensis* occurrence. Our best model predicted a probability of occurrence of *C*. *sonorensis* ranging from 0.45 to 0.60 when the precipitation in May was below or equal to 54 mm/month (up to 2 kg/m^2^/s*100,000). Above this threshold, a steep decrease in probability of occurrence was depicted (Fig 2).

### Model prediction under current conditions

**Fig 3:**
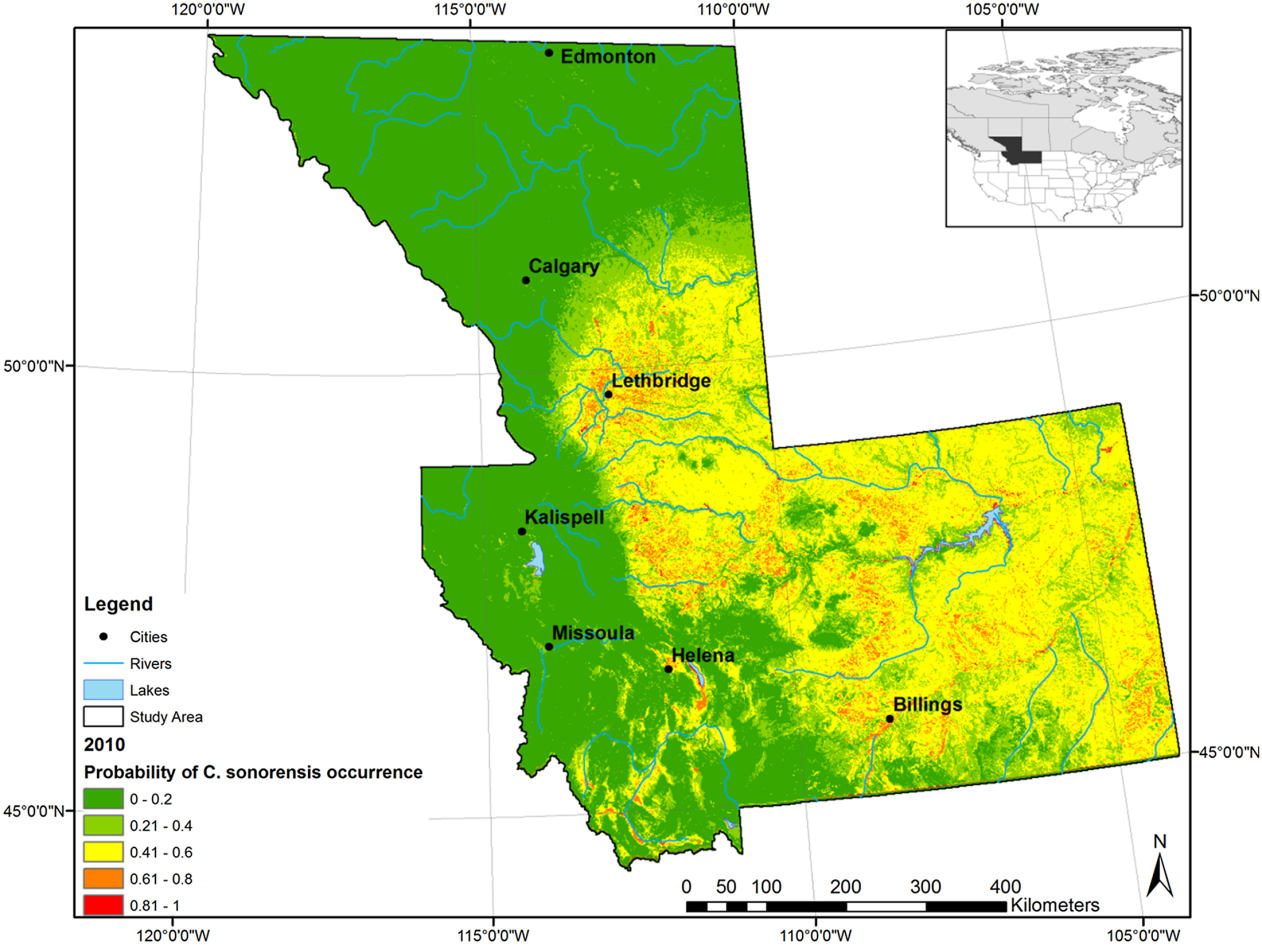
Probability distribution for *Culicoides sonorensis* in southern Alberta and Montana (2010) as estimated by the best performing model (B). Low probability classes are represented in green (0–40%) and high probability classes in orange (61–80%) and red (81–100%). The intermediate probability class (41–60%) is represented in yellow.

In the probability distribution map of *C*. *sonorensis* presence (Fig 3) predicted by the best performing model, most of the study area (45.9%; 286,530 km^2^) was predicted as at very low probability (*P* < 0.2), 19.9% (124,054 km^2^) at low (0.2 < *P* < 0.4), 29.7% (185,053 km^2^) at moderate (0.4 < *P* < 0.6) and the rest at moderate-high (0.6 < *P* < 0.8) and very high (*P* > 0.8) probability (4.2%, 26,070 km^2^, and 0.3%, 1,995 km^2^, respectively).

The probability map showed that areas close to the main rivers (i.e. Milk River, Missouri River and Yellowstone River) had a greater probability of *C*. *sonorensis* occurrence. The north-west part of the study area between Calgary and Edmonton was predicted to have lower probability (i.e. *P* < 0.2). Low probability of occurrence was also predicted along the Rocky Mountains and in correspondence to the highest peaks in both Alberta and Montana. The areas with greater probability (*P* ≥ 0.6) of occurrence of *C*. *sonorensis*, and therefore greater risk of disease transmission assuming the presence of suitable environmental conditions and susceptible hosts, were characterized by a number of environmental factors identified by MaxEnt model B (Tables 5 and 6). Mean monthly precipitation for May was 49.3 mm, with a range between 34.0 and 76.6 mm/month. Likewise, *VPD* for July ranged between 1.37–4.29 hPa, with a mean of 2.21. Grasslands made up half of those areas already at highest probability *Culicoides* occurrence under current conditions. Elevation ranged from 570 to 3,438 m with a mean of 990.2 m; mean standard elevation (2.96) described an environment at relatively lower elevations and smooth terrain. Collectively, these factors delineate the occurrence classes illustrated in orange and red in Fig 3.

**Table 5:** Values for best predictor variables identified by MaxEnt for areas currently above 0.6 probability threshold (60%).

	PMay (mm/month)	Esd (m)	Elev.(m)	*VPD* July (KPa)
**Minimum**	34.0	0.0	570	1.37
**Maximum**	76.6	253	3438	4.29
**Mean**	49.3	3	990	2.22
**Standard Deviation**	5.1	6	288	0.28

**Table 6:** Land cover percent area of landscape of total current (2010) area above 0.6 (60%) threshold category as identified by MaxEnt.

Land cover variable	Percent Area (%)
**Water bodies**	13.00
**Evergreen needleleaf forest**	0.02
**Open shrubland**	2.88
**Grassland**	50.00
**Cropland**	12.00
**Barren soil**	2.00
**Other**	20.00

### Model predictions under future conditions

**Fig 4:**
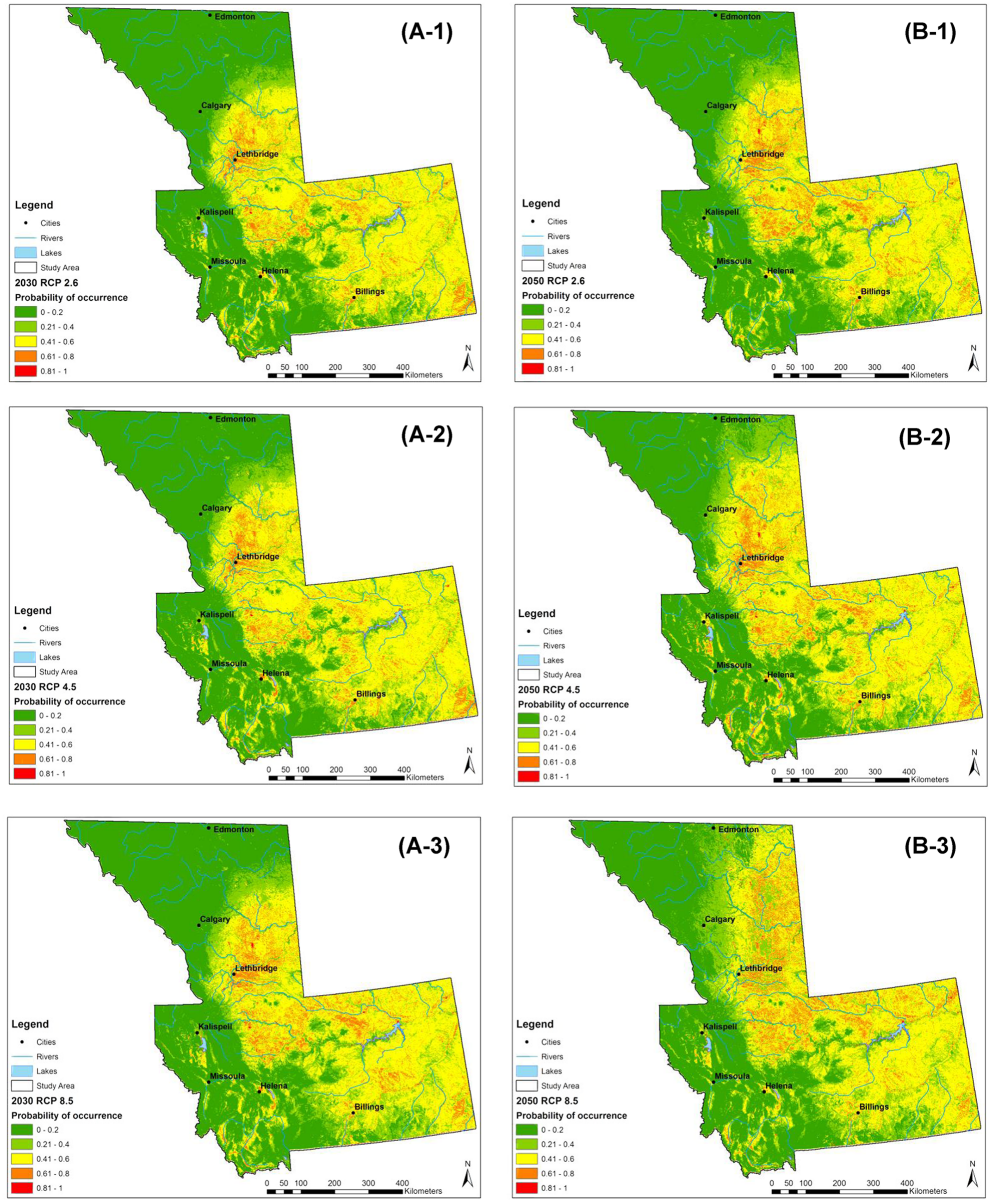
Probability distribution maps of *Culicoides sonorensis* under different projected climate change scenarios (Representative Concentration Pathways, RCP). A, left panel (2010–2030 scenarios): probability distribution map predicted by MaxEnt for 2010–2030 for each scenario. B, right panel (2030–2050 scenarios): probability distribution map predicted by MaxEnt for 2030–2050 for each RCP scenario. Each RCP is displayed (1) RCP 2.6; (2) RCP 4.5; (3) RCP 8.5.

The 2030 scenarios displayed an expansion of *C*. *sonorensis* occurrence with a notable advancement northwards (Fig 4(A-1), 4(A-2) and 4(A-3)). The same geographic trend was observed for the 2050 scenarios with an additional expansion identified north of the 53^rd^ parallel—previously considered the northern limit of BT distribution worldwide (Fig 4(B-1), 4(B-2) and 4(B-3)).

**Fig 5:**
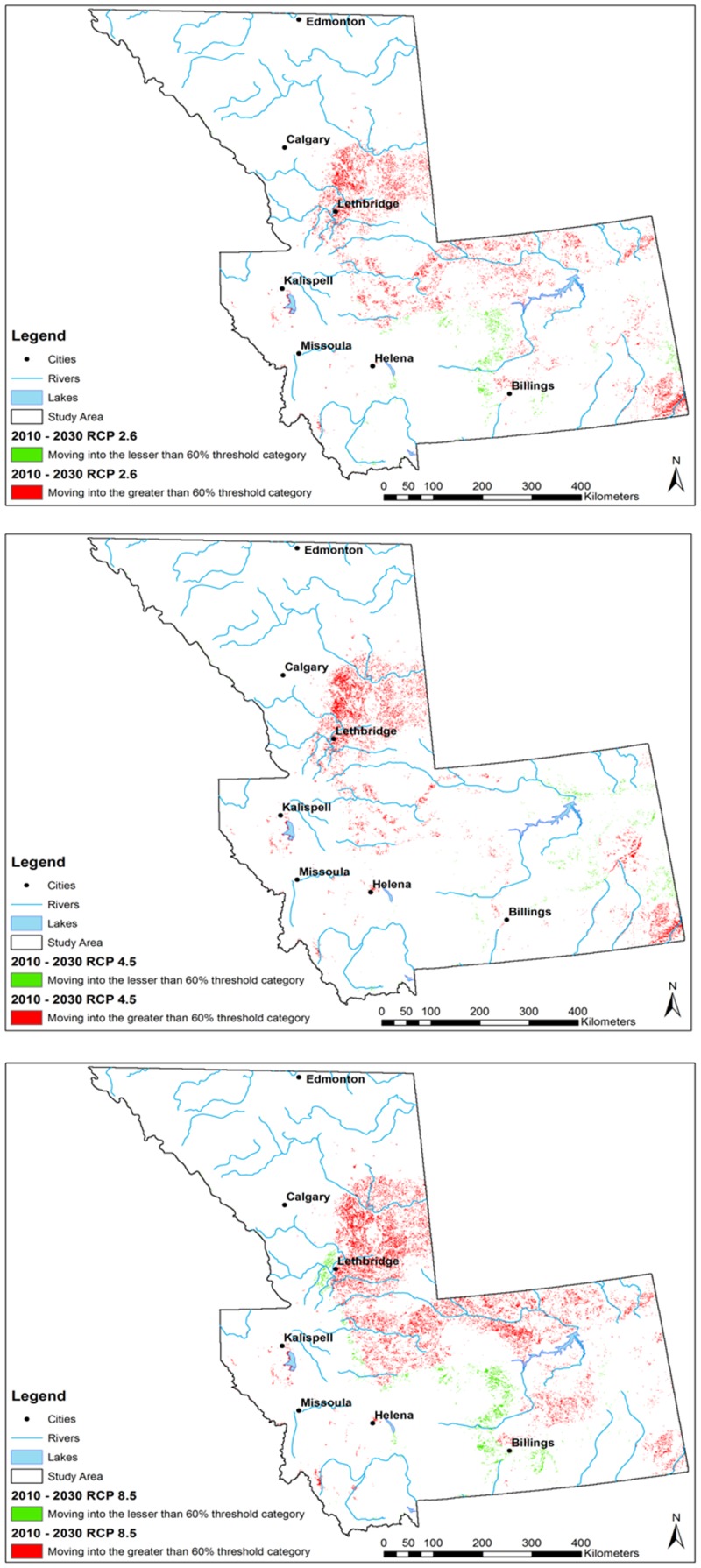
Maps of pixel change into and out of 0.6 threshold category (expressed as percentage) for *Culicoides sonorensis* occurrence probability as predicted with the Maximum Entropy algorithm under different climate change scenarios (Representative Concentration Pathways, RCP) projected for 2010–2030. Each RCP is displayed (A) RCP 2.6; (B) RCP 4.5; (C) RCP 8.5. Green represents those areas moving into lesser than 0.6 probability threshold category (expressed as percentage; 60%), red represents those areas moving into the greater than 60% probability threshold category for *Culicoides sonorensis* occurrence.

**Fig 6:**
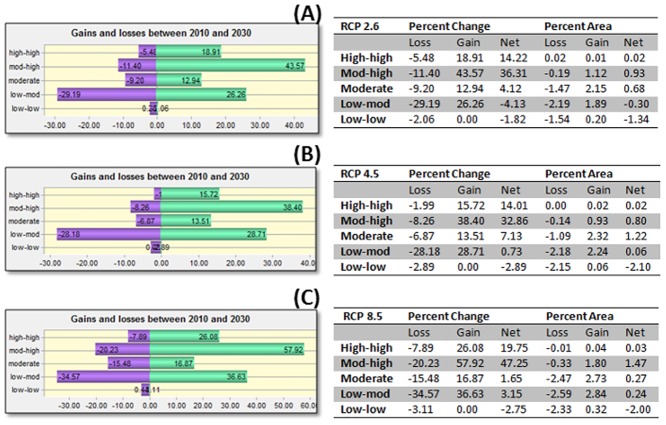
Change analysis into and out of 60% (0.6 probability) threshold category for *Culicoides sonorensis* occurrence probability as predicted with the Maximum Entropy algorithm under different climate change scenarios (Representative Concentration Pathways, RCP: RCP 2.6, RCP 4.5, RCP 8.5) projected for 2010–2030. Table represents both percent change and percent area of entire study area for loss, gain, and net change in pixels for each probability class category expressed as percentage (Low-low = 0–20%; low-mod = 21–40%; Moderate = 41–60%; Mod-high = 61–80%; and high-high = 81–100% probability of occurrence). Adjacent table visually indicates gains and losses (of percent change) for each class category. Percent change is defined by the number of pixels changed for a class divided by area of a class in later image, multiplied by one hundred. Percent area change is defined by the number of pixels changed for a class, divided by the total area of the land cover map, multiplied by one hundred.

By the 2030s, a 0.95% net gain in the percent area falling within the 61–100% probability classes (mod-high to high-high) was predicted under the RCP 2.6 scenario—increasing to an additional 1.5% under the more extreme RCP 8.5 scenario (Figs 5C and 6C).

**Fig 7:**
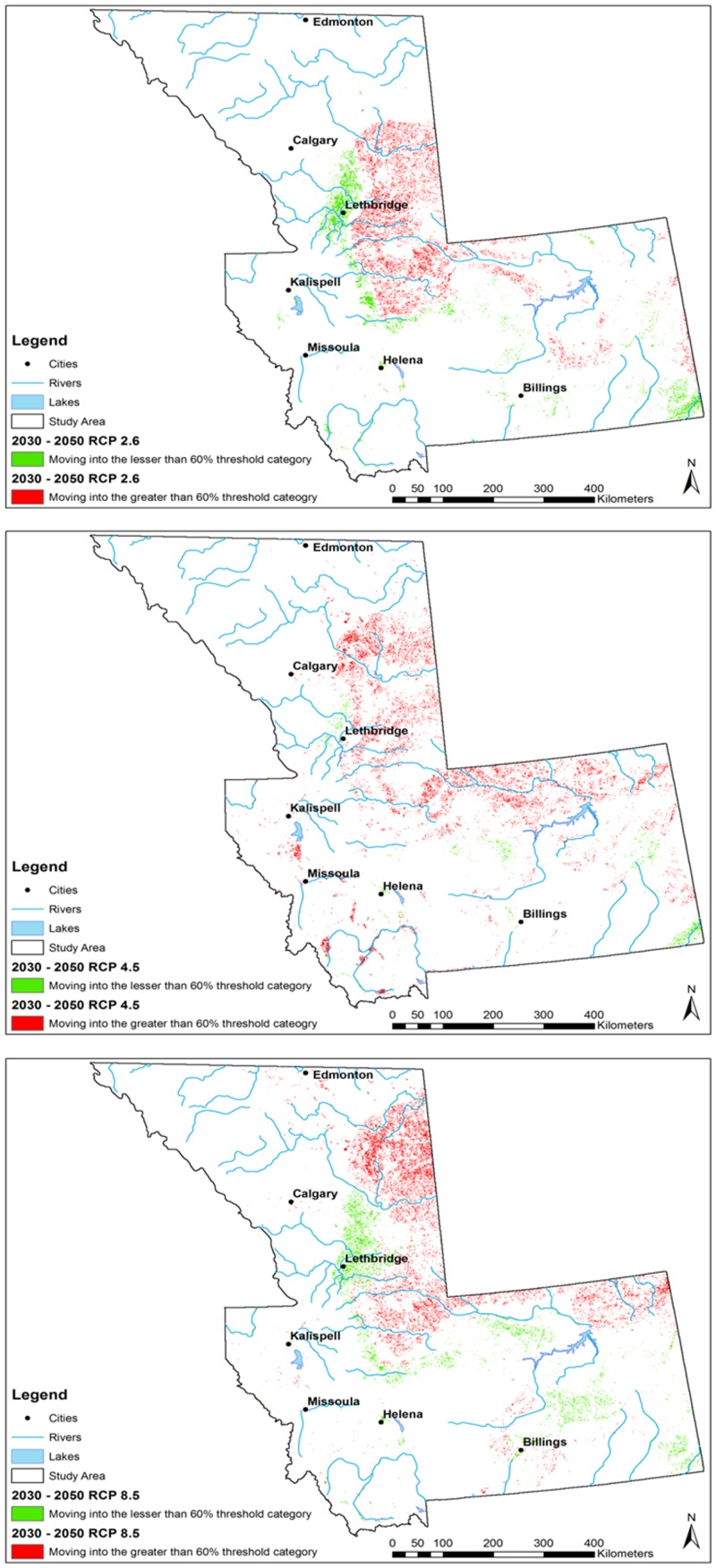
Maps of pixel change analysis into and out of 0.6 threshold category (expressed as percentage) for *Culicoides sonorensis* occurrence probability as predicted with the Maximum Entropy algorithm under different climate change scenarios (Representative Concentration Pathways, RCP) projected for 2030–2050. Each RCP is displayed (C) RCP 2.6; (B) RCP 4.5; (C) RCP 8.5. Green represents those areas moving into lesser than 60% probability threshold category, red represents those areas moving into the greater than 60% probability threshold category for *Culicoides sonorensis* occurrence.

**Fig 8:**
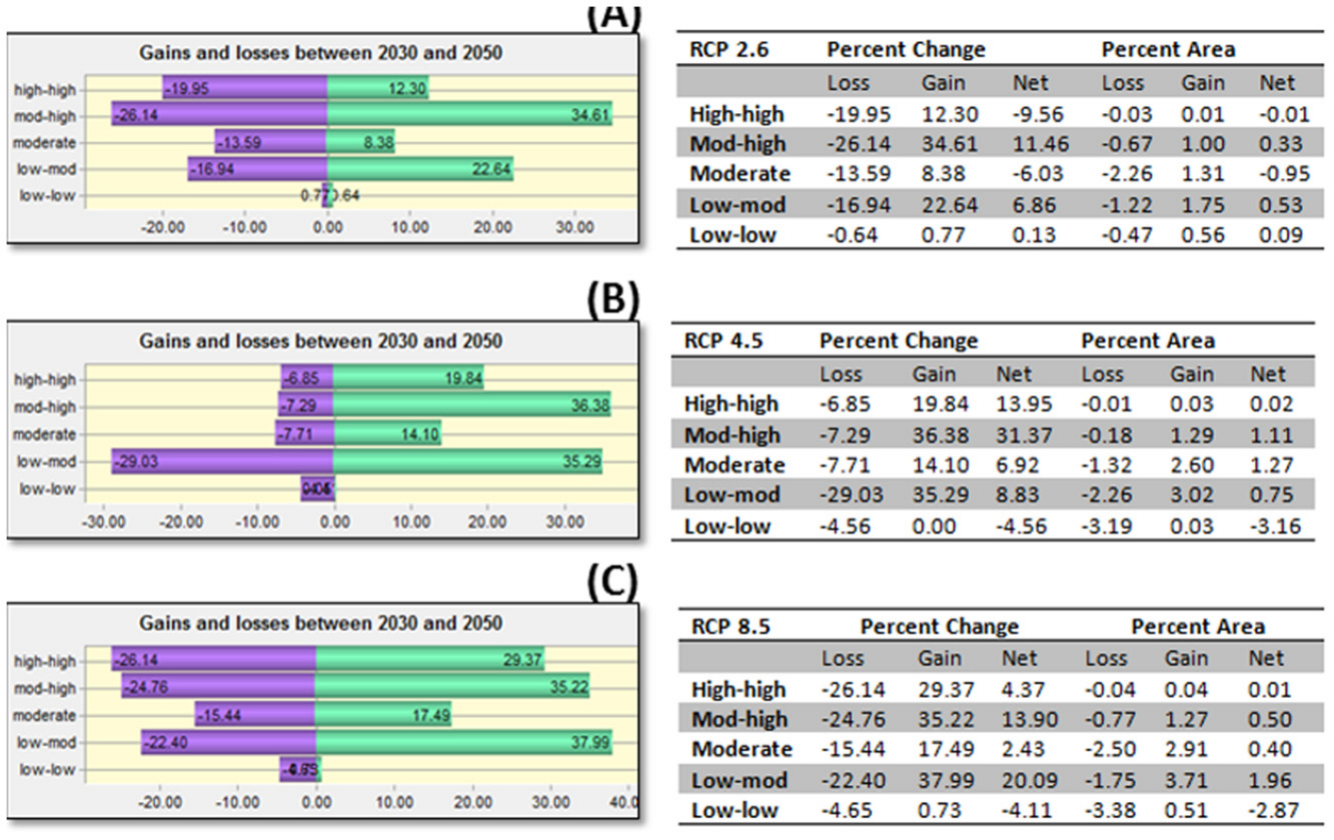
Change analysis into and out of 60% (0.6 probability) threshold category for *Culicoides sonorensis* occurrence probability as predicted with the Maximum Entropy algorithm under different climate change scenarios (Representative Concentration Pathways, RCP: RCP 2.6, RCP 4.5, RCP 8.5) projected for 2030–2050. Table represents both percent change and percent area of entire study area for loss, gain, and net change in pixels for each probability class category expressed as percentage (Low-low = 0–20%; low-mod = 21–40%; Moderate = 41–60%; Mod-high = 61–80%; and high-high = 81–100% probability of occurrence). Adjacent table visually indicates gains and losses (of percent change) for each class category. Percent change is defined by the number of pixels changed for a class divided by area of a class in later image, multiplied by one hundred. Percent area change is defined by the number of pixels changed for a class, divided by the total area of the land cover map, multiplied by one hundred.

In all scenarios, a net gain in the ≥ 0.6 probability threshold was predicted by 2030, while fewer regions were predicted to move into the lesser risk categories below the 0.6 threshold. An additional 0.3% of the study area was predicted to move into the ≥ 0.6 threshold probability of occurrence between 2030 and 2050 under the RCP 2.6 scenario (Figs 7A and 8A).

The model under the RCP 4.5 predicted a 1.1% additional increase in the probability of *C*. *sonorensis* within the entire study area between the 2030s and the 2050s. The RCP 8.5 pathway predicted an additional increase of 0.51% of area at greater risk for *C*. *sonorensis* occurrence by the 2050s. A slight reduction of *C*. *sonorensis* probability of occurrence is expected in central Montana by the mitigation (i.e. RCP 2.6) and the extreme (i.e. RCP 8.5) pathway in the 2030s (Fig 5A and 5C). Conversely, both the north and eastern regions of the study area is expected to see an increase in vector occurrence probability. By 2050 (Fig 7) a general expansion of the vector’s distribution is observed in all directions. During this time both the RCP 2.6 and RCP 8.5 scenarios forecast a reduction of probability of occurrence along the eastern foothills of the Rocky Mountains between Calgary, AB and Helena, MT. The Lethbridge area of southern Alberta is projected to decrease in vector presence probability, along with the central plains of Montana (Fig 7A and 7C).

In the majority of the projected climate change scenarios, a net gain was identified within the relatively higher risk classes, with the largest increase in those areas moving into the 0.61–0.8 probability class. Similarly, a net loss of the lowest probability class (0–0.2) was displayed in most scenarios (Figs 6 and 8).

## Discussion

With this study we identified large scale climate and environmental proxies to model the distribution of *C*. *sonorensis* at its northernmost latitudinal edge in south-central Alberta (Canada) and Montana (USA). Through these proxies we not only modeled the current distribution of *C*. *sonorensis*, but we also projected the future trends in distribution under three climate change scenarios. Our projections clearly indicated a shift in the northern edge of the distribution of the *Culicoides* vector in this region. Our results also highlighted the value of applying a maximum entropy presence-only modeling approach to distribution data at these latitudes (i.e. where climate change will have more impact [55] and therefore responses of the species might be greater) as they can be pivotal in the development of targeted surveillance activities for *Culicoides*-borne diseases.

### Large scale proxies of *Culicoides sonorensis* presence

Five models were developed to predict the distribution of *C*. *sonorensis* under current climate and environmental conditions. All of them had medium-high AUC values and therefore good performances. In previous studies bioclimatic variables like temperature and precipitation were selected as standard predictors for *Culicoides* spp. distribution modelling [56]. Our best model (Table 4) effectively predicted the current distribution of *C*. *sonorensis* using the correlation between *C*. *sonorensis* presence data and four environmental and climatic proxies (mean values of Vapour Pressure Deficit of July, mean values of Precipitation of May, standard deviation of Elevation and Land Cover). Statistical models such as MaxEnt, estimate the correlation between variables and species occurrence but cannot be used to infer causative relationships or the underlying biological mechanisms [57, 58]. However, correlative distribution models can still provide useful insights on current distribution when they are based on sound ecological knowledge and representative field data [58]. The main concerns with entomological field data are biases in the methodology used for collections and potential cross-correlation between predictor variables [59]. The entomological data used in this study geographically and environmentally covered the ecological range of *C*. *sonorensis* in Alberta and Montana. Although all these factors were considered in order to generate an accurate and simple model, the relationship between the best predictors (i.e. *VPD* of July, *P* of May, LC and E sd) and *C*. *sonorensis* occurrence is complex and hard to interpret.

Vapour pressure deficit of July provided the highest contribution to our predictions (54.5%). *VPD* was used to predict *Culicoides* spp. distribution in two European previous studies [32, 56] and it is considered to describe the combined effects of temperature and relative humidity on the survival of insects [40, 60]. In general, the increasing *VPD* values correspond to greater water losses from wet surfaces and in the case of *VPD* of July were correlated with *C*. *sonorensis* presence. A similar relationship calculated with annual values of *VPD*, was found by Wittmann *et al*. [56] when modeling *C*. *imicola* (Kieffer, 1913) distribution in Europe. These findings might be related to evaporation rates associated with water bodies that provide a suitable environment for immature stages of *C*. *sonorensis* [37]. Higher *VPD* values give higher evaporation rates, consequently higher salinity concentrations at potential *Culicoides* spp. breeding sites. This is considered to be a favourable condition for the development of *C*. *sonorensis* larvae [20, 21]. Additionally, in a laboratory study undertaken by Wittmann *et al*. [25] it was found that low *RH* (40%) resulted in an increased survival of *C*. *sonorensis* adults at high temperatures (25°C) compared to the same temperature at higher humidity rates (85%). The first RCP scenario corresponds to *VPD* values of 1.9 kPa, whereas the second corresponds to *VPD* values of 0.4 kPa. In our results *VPD* values above 1.9 kPa predicted a probability of *C*. *sonorensis* presence above 50%, whereas *VPD* values of 0.4 kPa corresponded to a minimal probability of *C*. *sonorensis* occurrence. Opposite trends were reported by Wittmann *et al*. [25] which might be due to the greater level of complexity for a large-scale species distribution model compared to a laboratory study.

According to our results, the presence of *C*. *sonorensis* was also inversely related to the mean precipitation of May. The highest probability of species occurrence corresponded to mean May *P* values of 54 mm/month and decreased after that threshold. These findings may be explained by the fact that heavy rainfall increases water levels at the breeding sites. Increased water levels can result in reduced salt concentrations and increased dilution of water contaminants (i.e. manure). These factors can inhibit the development of immature *C*. *sonorensis* [37]. Since Albertan *C*. *sonorensis* emerge in May [31], unfavorable conditions like heavy rainfalls at this point in time can affect population dynamics for the whole season [13, 24].

The standard deviation of elevation is a proxy for terrain complexity combining the effects of altitude and slope. *Culicoides sonorensis* had lower probability of occurrence where values of E sd were greater (i.e. more complex terrain). The areas with higher terrain variability such as slopes will retain less water, be at higher altitudes and characterized by colder temperatures resulting in their being unsuitable habitats for *C*. *sonorensis*.

Land cover classes such as water bodies, cropland and grassland were positively associated with the occurrence of *C*. *sonorensis*. This might be because suitable breeding sites are defined by plain terrains with water resources for natural or agricultural reasons and it is consistent with current knowledge on *C*. *sonorensis* land cover preferences [20]. The needle-leaf forest class is distributed along the Rocky Mountains and is strongly correlated to *C*. *sonorensis* absence, which might be due not only to shade over *C*. *sonorensis* potential habitat [61], but also to unfavorable altitude and related climatic factors (e.g. temperature).

### Current *Culicoides sonorensis* distribution

When considering the current distribution map predicted using MaxEnt, we observe that *C*. *sonorensis* has a low (*P* < 0.2) probability of occurrence in most of south-central Alberta. The risk of occurrence of *Culicoides*-borne disease in south-central Alberta, with current climatic and environmental conditions, is generally low with the exception of a higher probability of finding *C*. *sonorensis* in its southeastern corner [31]. The emergence of *Culicoides*-borne diseases depends on the presence of competent vectors, susceptible hosts and is influenced by climatic and environmental conditions. As a matter of fact, in September 2013 EHDV was isolated in Foremost (49.479 N, -111.440 W), southeastern Alberta, from wild ruminants after 50 years of virus absence in the Province [11]. In our model, this area was predicted to be at 0.55 probability of *C*. *sonorensis* occurrence, suggesting the need to consider the areas classified within the moderate probability of *C*. *sonorensis* occurrence class (0.41–0.6) as a threshold in which targeted surveillance for *Culicoides*-borne diseases is recommended.

In Montana, areas in the proximities of streams, lakes and dams are associated with a higher probability (> 0.60) of *C*. *sonorensis* occurrence. The model predictions are supported by the occurrence of disease outbreaks (e.g., ProMED archive number: 20110820.2529, 2011; [62, 63]) in the areas predicted to be at higher probability of *C*. *sonorensis* presence. On the other hand, the areas that experienced the most recent outbreaks of *Culicoides*-borne diseases were a primary target for vector surveillance and might have been over-sampled compared to areas considered at lower risk of disease occurrence.

### *Culicoides sonorensis* distribution under climate change scenarios

*Culicoides sonorensis* distribution projections into the 2030s and 2050s were built using the changes in July *VPD* and May precipitation, obtained for three different future climate change scenarios (RCP 2.6, RCP 4.5, RCP 8.5). New areas of the study region had a greater probability of *C*. *sonorensis* occurrence for both time frames, mainly at northern latitudes.

Moreover, the number of areas that were forecast to be at high probability of *C*. *sonorensis* occurrence (> 0.6) increased from the baseline period to the 2030s and from the 2030s to the 2050s for each RCP. The spatial distribution of these areas followed the northward directional shift of the northern distribution edge of *C*. *sonorensis*. This projection suggested that climatic and environmental conditions able to sustain vector presence are predicted to move northwards, delineating new northern limits above 53°N for *C*. *sonorensis* distribution. This suggests that there might be a greater risk of *C*. *sonorensis*-transmitted diseases occurring in the overall area, with a clear trend in the expansion of the vector distribution at northern latitudes. The presence of a competent vector is a *conditio sine qua non* for disease transmission, but it is not sufficient to allow transmission. Other components of the pathogen-vector life cycle have to be considered, such as the vectorial capacity [23, 64] and host availability, and should be estimated in order to assess disease transmission risk.

The projections described neglect to consider direct impacts of land cover change on species distribution. In application of the models to future climate change scenarios, we have assumed that these fields are unchanged relative to the baseline period, 1991–2010. Changes in land cover can be expected over the next 40 years, but are expected to be more local than regional over this time frame. This is nonetheless an uncertainty in our projections, and our results and conclusions are restricted to the potential effects of climate change on *C*. *sonorensis* distribution. The RCP climate change scenarios do include land-cover changes, but we do not map these onto LC indices in this study.

The reliability of species distribution models under future climatic conditions depends on the quality of the distribution model built under current climatic conditions and the data used to generate it. Our approach (using a maximum entropy algorithm) assumes that the relationship between variables and species remains the same through time; therefore, it is essential that the selected predictor variables are biologically meaningful and presence points fairly represent the whole environmental range in which *C*. *sonorensis* exists. Even if all these precautions were taken into consideration, modeling species distributions under future climate can generate useful but still uncertain results [65].

## Conclusions

Bluetongue and EHD occur sporadically in Western Canada [11, 14] raising concerns of disease incursion and transmission in the region. A key outcome from the work outlined in this paper is the identification of mean values of Vapour Pressure Deficit of July, mean values of Precipitation of May, standard deviation of Elevation and Land Cover classes as potential ecological proxies of *C*. *sonorensis* at a regional scale and their usage to predict *C*. *sonorensis* present and future northernmost distribution edge. The maps generated using the maximum entropy approach can be used to inform current and future vector surveillance programs, targeting areas within the known limits of *C*. *sonorensis* geographical occurrence under current and future climatic conditions. This has the potential to allow a targeted and informed approach to reduce the costs of an extensive surveillance plan while enhancing its efficacy. Targeted vector surveillance, possibly extended to neighboring provinces that have previously experienced Orbiviruses incursions, would also enhance model performance and consequently the quality of preparedness plans. Furthermore, *C*. *sonorensis* probability distribution maps can be used as a starting point in the assessment of disease transmission risk, given the availability of information on vectorial capacity.

## Supporting Information

S1 Fig Best predictors for *C*. *sonorensis* distribution modeling shown as geographic layers: land cover. (TIF)

S2 Fig Best predictors for *C*. *sonorensis* distribution modeling shown as geographic layers: July vapour pressure deficit, *VPD*. (TIF)

S3 Fig Best predictors for *C*. *sonorensis* distribution modeling shown as geographic layers: precipitation of May. (TIF)

S4 Fig Best predictors for *C*. *sonorensis* distribution modeling shown as geographic layers: standard deviation of elevation. (TIF)

S1 Table Presence absence data *Culicoides sonorensis* in Southern Alberta (Canada) and Montana (USA) from 2002 till 2012. (DOCX)

S2 Table Land cover type 1 classification scheme of the International Geosphere Biosphere Programme (IGBP) and classification scheme used in the present study. (DOCX)
