# Supplementary figures and images for: Modelling the Northward Expansion of Culicoides sonorensis (Diptera: Ceratopogonidae) under Future Climate Scenarios
Source: PLoS One. 2015 Aug 24;10(8):e0130294. doi: 10.1371/journal.pone.0130294 (PMC4547716; doi:10.1371/journal.pone.0130294)

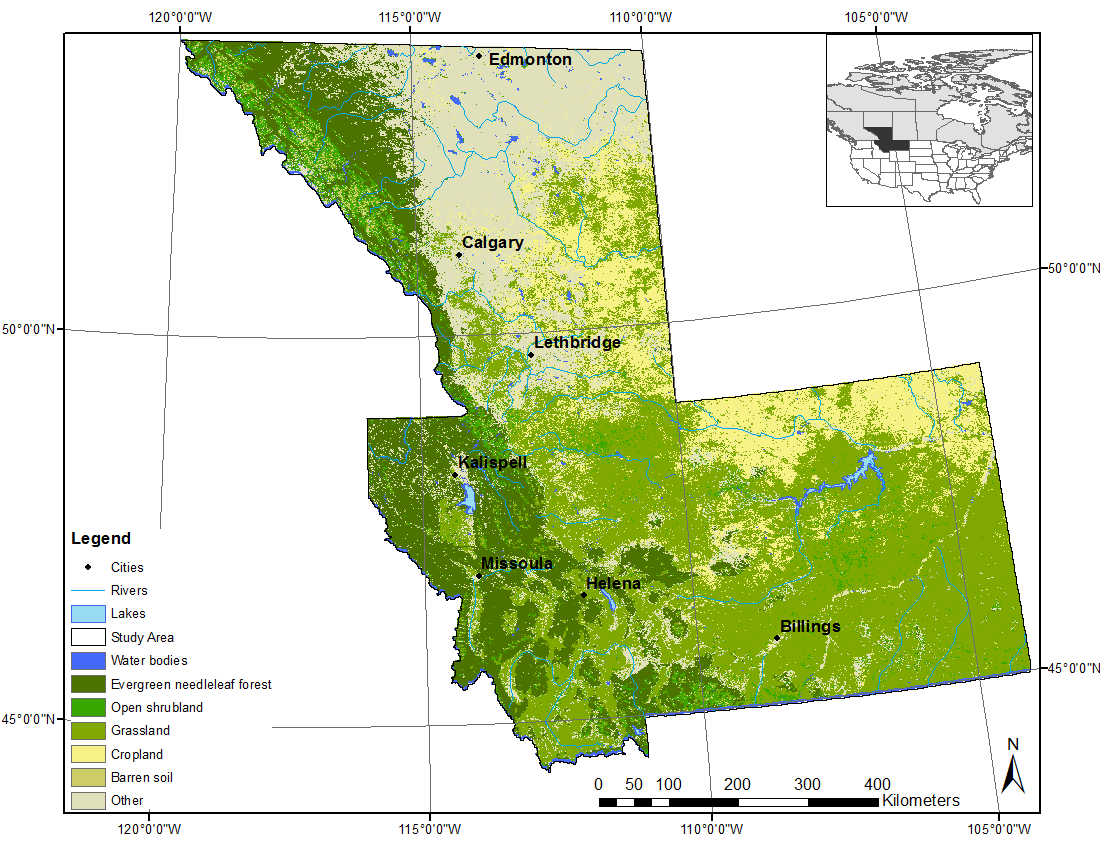

Supplement: S1 Fig — (TIF) [file pone.0130294.s001.tif]

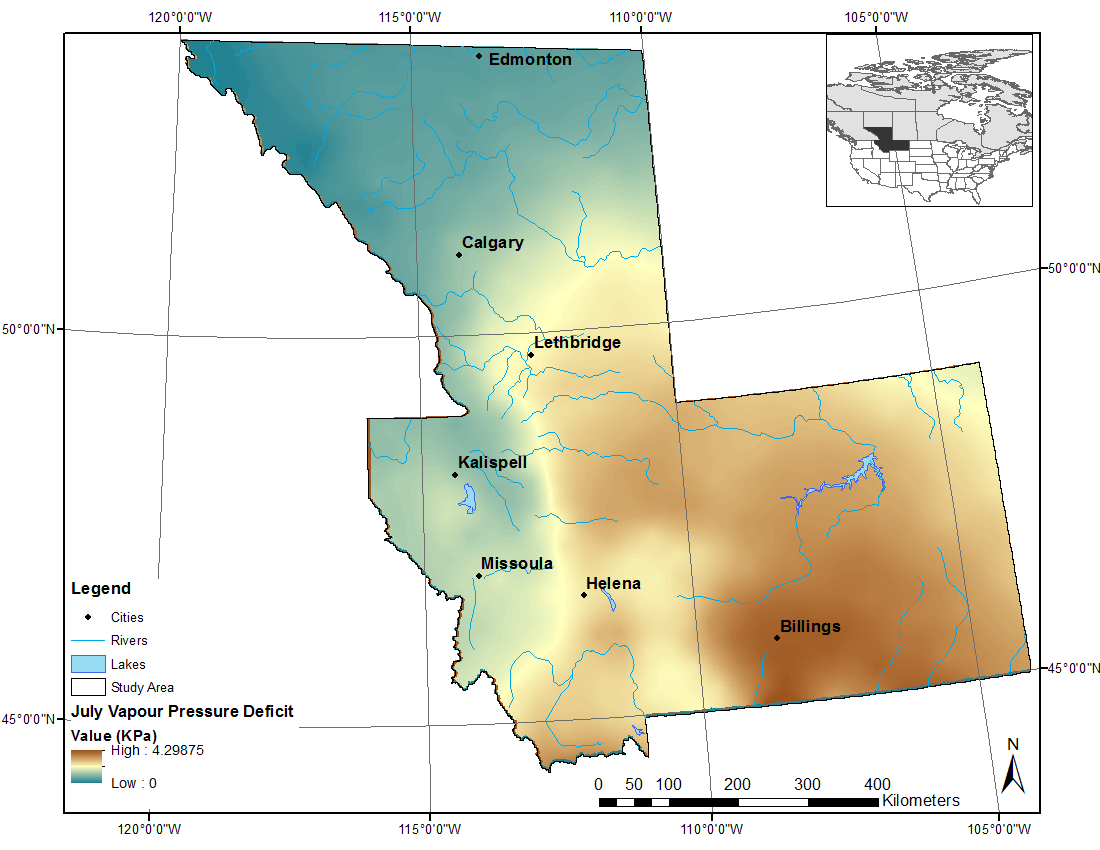

Supplement: S2 Fig — (TIF) [file pone.0130294.s002.tif]

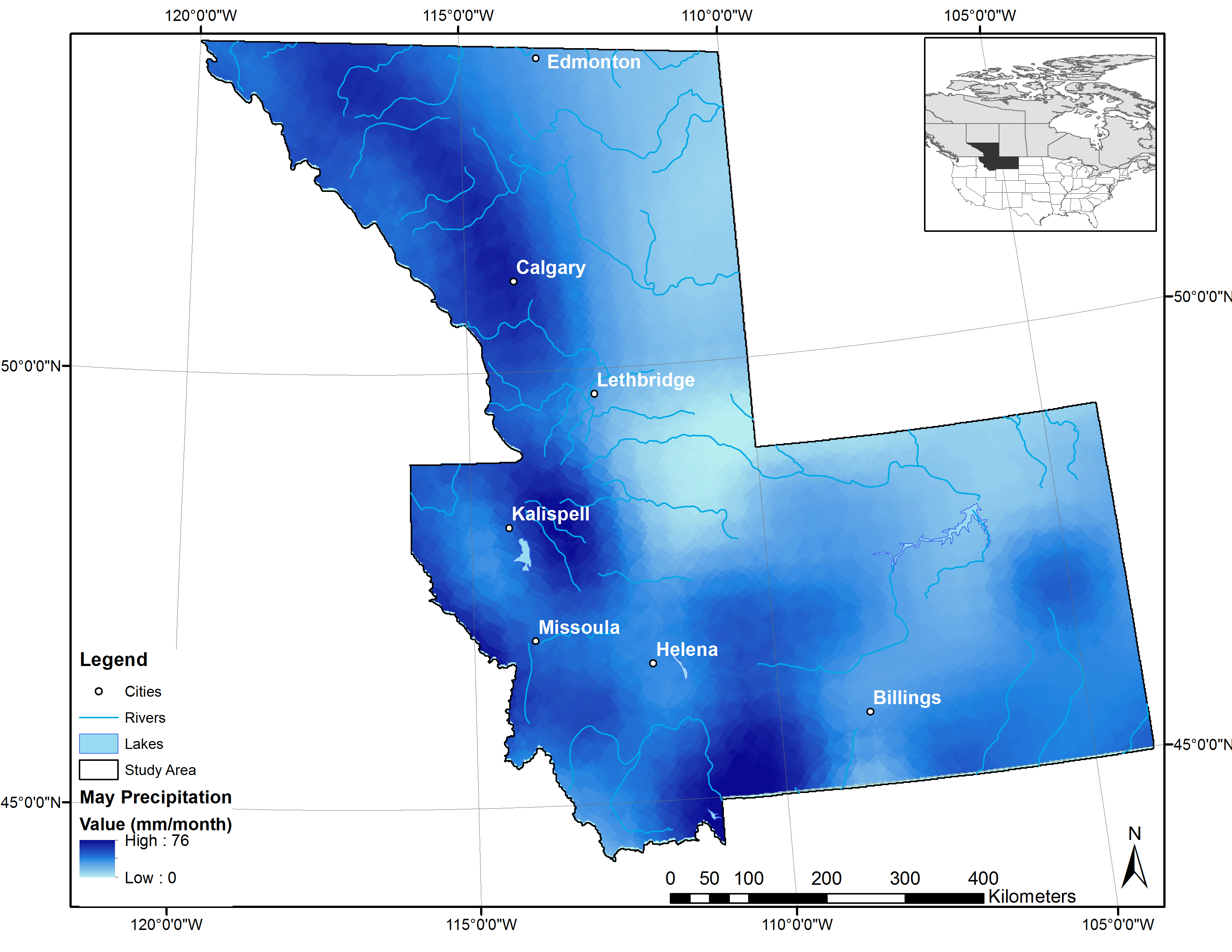

Supplement: S3 Fig — (TIF) [file pone.0130294.s003.tif]

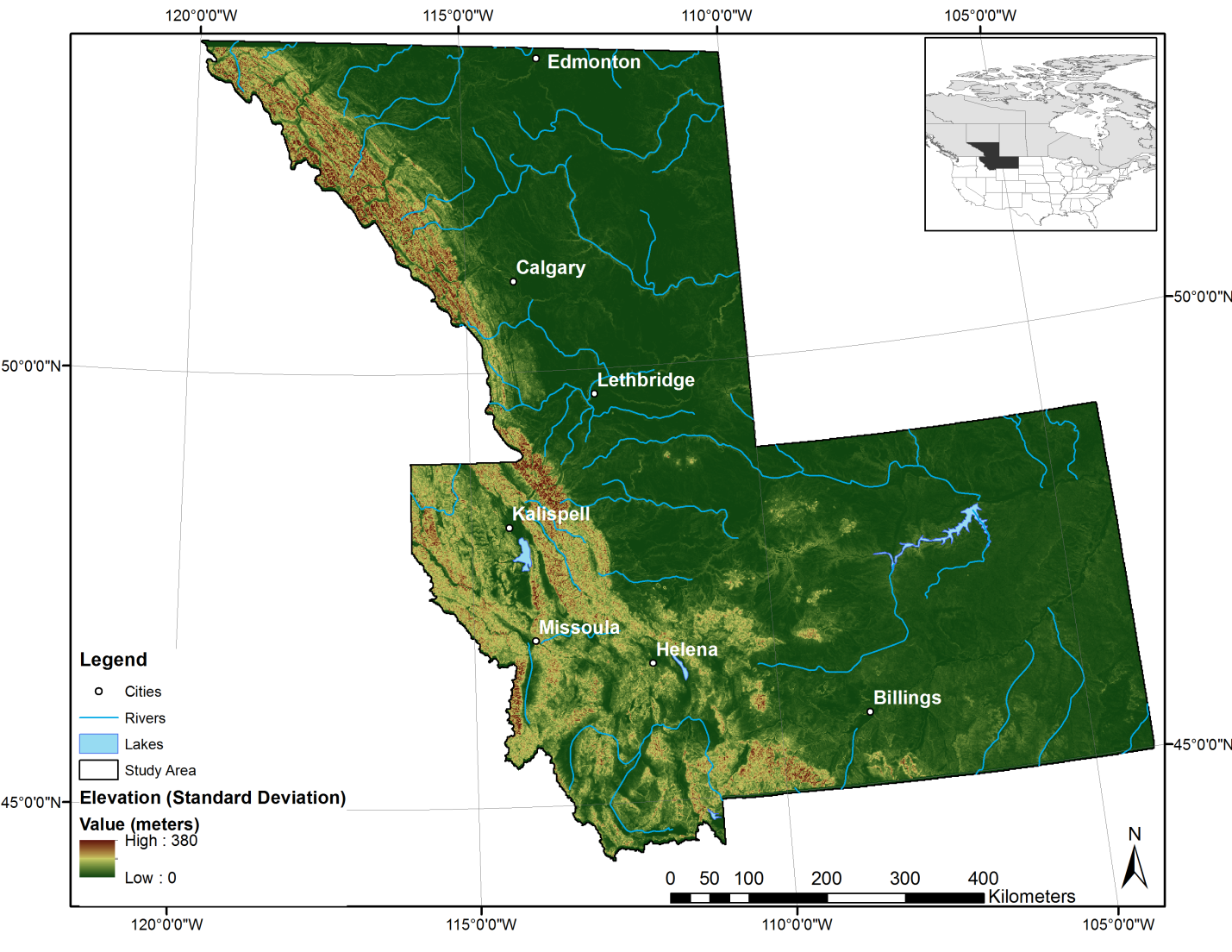

Supplement: S4 Fig — (TIF) [file pone.0130294.s004.tif]
