# Supplementary material for: Modelling the Northward Expansion of Culicoides sonorensis (Diptera: Ceratopogonidae) under Future Climate Scenarios
Source: PLoS One. 2015 Aug 24;10(8):e0130294. doi: 10.1371/journal.pone.0130294 (PMC4547716; doi:10.1371/journal.pone.0130294)
Supplement: S1 Table — (DOCX) [file pone.0130294.s005.docx]

Supporting Information Text S1. Presence absence data *Culicoides sonorensis* in Southern Alberta (Canada) and Montana (USA) from 2002 till 2012.

| Lat | Long | Presence (1)/Absence (0) |
| --- | --- | --- |
| 50.61377 | -111.95376 | 0 |
| 51.29418 | -110.80150 | 1 |
| 51.94563 | -112.96613 | 0 |
| 51.65729 | -114.56891 | 0 |
| 50.15650 | -112.36681 | 1 |
| 49.38657 | -111.10493 | 1 |
| 49.63486 | -113.30481 | 1 |
| 51.39933 | -111.86034 | 0 |
| 50.54303 | -110.28381 | 1 |
| 51.82350 | -110.84348 | 0 |
| 49.95189 | -112.64092 | 1 |
| 53.04522 | -111.52340 | 0 |
| 53.04521 | -111.57682 | 0 |
| 52.46062 | -113.78107 | 0 |
| 52.44712 | -113.79099 | 0 |
| 51.36905 | -112.19910 | 0 |
| 49.69123 | -112.76990 | 1 |
| 50.72190 | -113.96522 | 0 |
| 49.12555 | -110.46897 | 1 |
| 49.12960 | -110.47160 | 1 |
| 51.32567 | -110.42521 | 0 |
| 49.98317 | -112.76244 | 1 |
| 49.95416 | -112.90226 | 1 |
| 51.85278 | -112.72112 | 0 |
| 51.59212 | -110.66210 | 0 |
| 50.19282 | -113.89542 | 0 |
| 49.46261 | -112.52475 | 1 |
| 52.04122 | -112.12368 | 0 |
| 50.01360 | -112.17403 | 1 |
| 49.32388 | -112.16582 | 1 |
| 48.03050 | -110.83780 | 1 |
| 48.24460 | -110.87970 | 1 |
| 47.91330 | -112.36820 | 1 |
| 48.72710 | -112.39180 | 1 |
| 48.26420 | -110.99300 | 0 |
| 46.36430 | -105.92170 | 1 |
| 46.39260 | -105.62350 | 1 |
| 46.42100 | -105.70120 | 1 |
| 48.03290 | -106.66900 | 1 |
| 48.52990 | -106.70610 | 1 |
| 48.40560 | -106.67700 | 1 |
| 48.14970 | -107.87300 | 1 |
| 46.34570 | -104.41080 | 1 |
| 45.60580 | -107.02730 | 1 |
| 45.16400 | -105.48450 | 1 |
| 45.96290 | -105.70480 | 1 |
| 46.83680 | -104.20710 | 1 |
| 45.69220 | -111.33050 | 0 |
| 45.79330 | -111.31430 | 0 |
| 45.83060 | -111.46580 | 0 |
| 47.70920 | -105.08140 | 1 |
| 46.33120 | -106.52690 | 1 |
| 47.18270 | -110.00070 | 1 |
| 45.92480 | -108.19700 | 1 |
| 47.40470 | -109.92230 | 1 |
| 46.93520 | -109.77770 | 1 |
| 46.92500 | -109.77690 | 1 |
| 48.39830 | -107.73200 | 1 |
| 48.40000 | -107.71660 | 1 |
| 48.42860 | -107.70060 | 1 |
| 47.43690 | -109.69820 | 1 |
| 45.78800 | -108.13600 | 1 |
| 46.38100 | -105.89200 | 1 |
| 46.46000 | -105.79600 | 1 |
| 47.09900 | -106.71300 | 1 |
| 47.35400 | -106.45200 | 1 |
| 47.03100 | -107.87300 | 0 |
| 45.98000 | -107.94700 | 1 |
| 45.21100 | -112.67400 | 0 |
| 46.16700 | -111.56900 | 0 |
| 46.65900 | -113.13700 | 0 |
| 46.70100 | -111.96100 | 0 |
| 46.79900 | -105.32500 | 1 |
| 46.91100 | -113.01200 | 0 |
| 47.45100 | -114.14800 | 0 |
| 47.71200 | -104.18700 | 1 |
| 48.56100 | -109.71100 | 0 |
| 45.53500 | -111.29500 | 1 |
