# Supplementary material for: Modelling the Northward Expansion of Culicoides sonorensis (Diptera: Ceratopogonidae) under Future Climate Scenarios
Source: PLoS One. 2015 Aug 24;10(8):e0130294. doi: 10.1371/journal.pone.0130294 (PMC4547716; doi:10.1371/journal.pone.0130294)
Supplement: S2 Table — (DOCX) [file pone.0130294.s006.docx]

Supporting Information Text S2. Land cover type 1 classification scheme of the International Geosphere Biosphere Programme (IGBP) and classification scheme used in the present study.

| **Class** | **IGBP (Type 1)** | **Description** | **Land cover classification for the present study** |
| --- | --- | --- | --- |
| 0 | Water Bodies | Oceans, seas, lakes, reservoirs, and rivers. Can be either fresh or salt water bodies. | Water Bodies (class 1) |
| 1 | Evergreen Needleleaf forest | Lands dominated by trees with a percent canopy cover >60% and height exceeding 2 meters. Almost all trees remain green all year. Canopy is never without green foliage. | Evergreen Needleleaf forest (class 2) |
| 2 | Evergreen Broadleaf forest | Lands dominated by trees with a percent canopy cover >60% and height exceeding 2 meters. Almost all trees remain green all year. Canopy is never without green foliage. | Other (class 7) |
| 3 | Deciduous Needleleaf forest | Lands dominated by trees with a percent canopy cover >60% and height exceeding 2 meters. Consists of seasonal needleleaf tree communities with an annual cycle of leaf-on and leaf-off periods. | Other (class 7) |
| 4 | Deciduous Broadleaf forest | Lands dominated by trees with a percent canopy cover >60% and height exceeding 2 meters. Consists of seasonal broadleaf tree communities with an annual cycle of leaf-on and leaf-off periods. | Other (class 7) |
| 5 | Mixed forest | Lands dominated by trees with a percent canopy cover >60% and height exceeding 2 meters. Consists of tree communities with interspersed mixtures or mosaics of the other four forest cover types. None of the forest types exceeds 60% of landscape. | Other (class 7) |
| 6 | Closed shrublands | Lands with woody vegetation less than 2 meters tall and with shrub canopy cover is >60%. The shrub foliage can be either evergreen or deciduous. | Other (class 7) |
| 7 | Open shrublands | Lands with woody vegetation less than 2 meters tall and with shrub canopy cover is between 10-60%. The shrub foliage can be either evergreen or deciduous. | Shrublands (class 3) |
| 8 | Woody savannas | Lands with herbaceous and other understorey systems, and with forest canopy cover between 30-60%.The forest cover height exceeds 2 meters. | Other (class 7) |
| 9 | Savannas | Lands with herbaceous and other understorey systems, and with forest canopy cover between 10-30%.The forest cover height exceeds 2 meters. | Other (class 7) |
| 10 | Grasslands | Lands with herbaceous types of cover. Tree and shrub cover is less than 10%. | Grasslands (class 4) |
| 11 | Permanent wetlands | Lands with a permanent mixture of water and herbaceous or woody vegetation that cover extensive areas. The vegetation can be present in either salt, brackish, or fresh water. | Other (class 7) |
| 12 | Croplands | Lands covered with temporary crops followed by harvest and a bare soil period | Croplands (class 5) |
| 13 | Urban and built-up | Land covered by buildings and other man-made structures. | Other (class 7) |
| 14 | Cropland/Natural vegetation mosaic | Lands with a mosaic of croplands, forest, shrublands, and grasslands in which no one component comprises more than 60% of the landscape. | Other (class 7) |
| 15 | Snow and ice | Lands under snow and/or ice cover throughout the year. | Other (class 7) |
| 16 | Barren or sparsely vegetated | Lands exposed soil, sand, rocks, or snow and never has more than 10% vegetated cover during any time of the year. | Barren or sparsely vegetated (class 6) |
| 254 | Unclassified |  |  |
| 255 | Fill Value |  |  |
